# The chemopreventive effect of *Ginkgo biloba *and *Silybum marianum *extracts on hepatocarcinogenesis in rats

**DOI:** 10.1186/1475-2867-11-38

**Published:** 2011-10-31

**Authors:** Hala O El Mesallamy, Nadia S Metwally, Mahmoud S Soliman, Kawkab A Ahmed, Mai M Abdel Moaty

**Affiliations:** 1 Faculty of Pharmacy, Biochemistry Department, Ain Shams University, Abbassia, Cairo 11566, Egypt; 2 Therapeutical Chemistry Department, Pharmaceutical and Drug Industries Research Division, National Research Centre (NRC), Tahrir st., Dokki, Giza, Egypt; 3 Faculty of Veterinary Medicine, Pathology Department, Cairo University, Giza, Egypt

**Keywords:** Hepatocellular carcinoma, *Ginkgo biloba*, *Silybum marianum*, N-nitrosodiethylamine, Antioxidants, Vascular endothelial growth factor, Comet assay

## Abstract

**Background/objective:**

This study was designed to evaluate the potential chemopreventive activities of *Ginkgo biloba *extract (EGb) and *Silybum marianum *extract (silymarin) against hepatocarcinogenesis induced by N-nitrosodiethylamine (NDEA) in rats.

**Methods:**

Rats were divided into 6 groups. Group 1 served as normal control rats. Group 2 animals were intragastrically administrated NDEA at a dose of 10 mg/kg five times a week for 12 weeks to induce hepatocellular carcinoma (HCC). Groups 3 and 4 animals were pretreated with silymarin and EGb respectively. Groups 5 and 6 animals were posttreated with silymarin and EGb respectively. The investigated parameters in serum are alanine aminotransferase (ALT), aspartate aminotransferase (AST), gamma glutamyltransferase (GGT) and vascular endothelial growth factor (VEGF). The investigated parameters in liver tissue are malondialdehyde (MDA), glutathione (GSH), superoxide dismutase (SOD), glutathione peroxidase (GPx), glutathione reductase (GR) and comet assay parameters.

**Results:**

In NDEA group, MDA level was elevated with subsequent decrease in GSH level and SOD, GPx and GR activities. In addition, NDEA group revealed a significant increase in serum ALT, AST and GGT activities and VEGF level. Furthermore, NDEA administrated animals showed a marked increase in comet assay parameters. These biochemical alterations induced by NDEA were confirmed by the histopathological examination of rat livers intoxicated with NDEA that showed an obvious cellular damage and well differentiated HCC.

In contrast, silymarin+NDEA treated groups (3&5) and EGb+NDEA treated groups (4&6) showed a significant decrease in MDA level and a significant increase in GSH content and SOD, GPx and GR activities compared to NDEA group. Silymarin and EGb also beneficially down-regulated the increase in serum ALT, AST, GGT activities and VEGF level induced by NDEA. In addition, silymarin and EGb significantly decreased comet assay parameters. Histopathological examination of rat livers treated with either silymarin or EGb exhibited an improvement in the liver architecture compared to NDEA group.

**Conclusions:**

The obtained findings suggested that silymarin and EGb may have beneficial chemopreventive roles against hepatocarcinogenesis through their antioxidant, antiangiogenic and antigenotoxic activities.

## Introduction

Hepatocellular carcinoma is the fifth most common cancer worldwide and the third most common cause of cancer mortality with a continuously increasing incidence annually [1]. The major known risk factors for HCC are viral (chronic hepatitis B and hepatitis C), toxic (alcohol and aflatoxins), metabolic (diabetes and non alcoholic fatty liver disease, hereditary haemochromatosis), immune related (primary biliary cirrhosis and autoimmune hepatitis), food additives, environmental and industrial toxic chemicals and air and water pollutants [2]. In Egypt, between 1993 and 2002, there was an almost two-fold increase in HCC amongst chronic liver patients [3]. It has been found that hepatitis C virus (HCV) and schistosomiasis play a major role in the development of HCC in Egypt where the highest prevalence of HCV in the world which has been attributed to previous public health eradication schemes for schistosomiasis [4,5]. Although there are many strategies for the treatment of HCC [6], its therapeutic outcome remains very poor. Therefore, preventive strategies are of paramount importance and need to be actively explored in order to reduce the incidence of this disease. The reduced cancer risk and lack of toxicity associated with high intake of natural products suggest that specific concentrations of phytochemicals from these plant sources may produce cancer chemopreventive effects without causing significant levels of toxicity. These natural products are believed to suppress the inflammatory process that lead to neoplastic transformation, hyperproliferation, promotion and progression of carcinogenic process and angiogenesis [7].

Silymarin, a standardized extract from *Silybum marianum *(milk thistle; a medicinal plant), has been extensively studied and has shown anticancer efficacy against various cancer sites [8,9]. This has been attributed to that silymarin interfers with the expression of cell cycle regulators and proteins involved in apoptosis. Additionally, silymarin has antioxidant properties and anti-metastatic activity [10]. Several studies have shown that silymarin has tumor suppressive effect on hepatocarcinogenesis [11-13].

*Ginkgo biloba*, a unique tree, is one of the best known examples of a living fossil. Ginkgo leaf extract has powerful anti-cancer properties through its antioxidant, gene-regulatory and antiangiogenic properties [14]. *Ginkgo biloba *extract may regulate cell proliferation and induce apoptosis of human hepatocellular cell lines: HepG2, Hep3B, and SMMC-7721 cells, therefore it may have protective effects against hepatocarcinogenesis [15,16].

In the present study, we investigated the chemopreventive effects of *Ginkgo biloba *and *Silybum marianum *extracts as antioxidant, antiangiogenic and antigenotoxic substances against HCC induced by NDEA in experimental animals. This may be promising at stopping hepatocarcinogenesis, delaying its progress, minimizing the damage to liver cells or reducing its complications.

## Materials and methods

### Animals

Healthy male Wistar albino rats weighing 100-120 g supplied from the animal house of National Research Center (Dokki, Giza, Egypt) were used for this study. Animals were housed in cages under proper environmental conditions at room temperature 22-24°C and 12 h light/dark cycle and fed with a commercial pellet diet (Wadi El Kabda Co., Cairo, Egypt). The animals had free access to water. The animals were acclimatized to the laboratory conditions for two weeks before beginning the experiment. The experiment continued for 13 weeks on which constant weight of diet was given for each rat. All the experiments were designed and conducted according to the ethical norms approved by the Ethical Committee of National Research Center.

### Chemicals

N-nitrosodiethylamine and ethidium bromide were purchased from Sigma-Aldrich (St. Louis, MO, USA), vascular endothelial growth factor ELISA kit was purchased from RayBiotech, Inc. (Milford, MA, USA), AST and ALT kits were purchased from Stanbio laboratory (Boerne, TX, USA) and GGT kit was purchased from Centronic GmbH (Wartenberg, Germany). Silymarin was purchased from ACAPI Co. (Cairo, Egypt). Silymarin consists of 80% w/w of silybin with smaller amounts of isosilybin, dihydrosilybin, silydianin, and silychristin. EGb was purchased from EMA Pharm Co. (Cairo, Egypt). EGb consists of approximately 24% flavone glycosides (quercetin, kaempferol and isorhamnetin), 6% terpene trilactones (ginkgolides A, B and C, bilobalide) and less than 5 ppm ginkgolic acid. All the other chemicals used were of high analytical grade and were purchased locally.

### Experimental design

**Figure 1:**
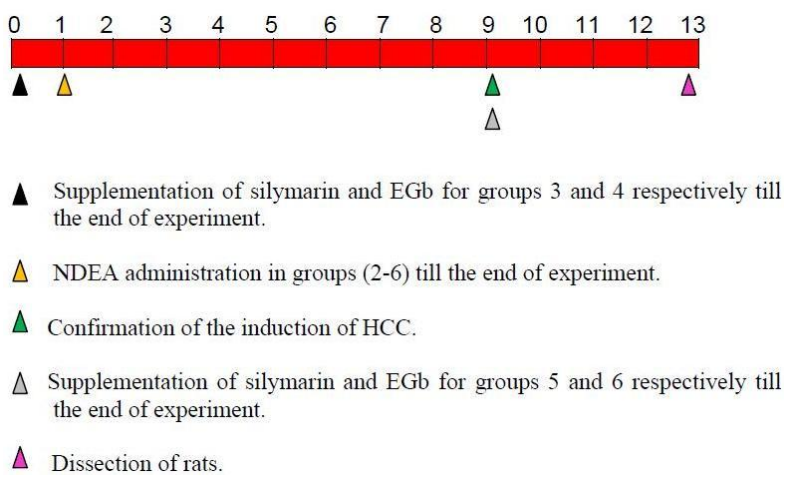
**The experimental design diagram**.

The experimental animals were divided into six groups (as shown in figure 1); each group was comprised of eight animals except group 2 which was comprised of 12 animals (4 rats were used for the confirmation of the induction of HCC at the end of 9^th ^week of the experiment by the histopathological examination of rat livers). Group 1: Normal control rats fed with standard diet for 13 weeks. Group 2: Rats were induced with HCC by NDEA received intragastrically at a dose of 10 mg/kg body weight 5 times a week for 12 weeks [17]. Group 3: Rats pretreated with silymarin (100 mg/kg body weight daily) received intragastrically one week before the administration of NDEA and continued till the end of the experiment (i.e. 13 weeks) [18]. Group 4: Rats pretreated with EGb (100 mg/kg body weight daily) received intragastrically one week before the administration of NDEA and continued till the end of the experiment [19]. Group 5: Rats posttreated with silymarin (100 mg/kg body weight daily) received intragastrically for 4 weeks after the administration of NDEA for 8 weeks and continued till the end of experiment [18]. Group 6: Rats posttreated with EGb (100 mg/kg body weight daily) received intragastrically for 4 weeks after the administration of NDEA for 8 weeks and continued till the end of experiment [19]. At the end of experimental period, the animals were fasted overnight then subjected to mild ether anesthesia and blood samples were collected. The blood was allowed to coagulate and centrifuged at 3000 rpm for 15 minutes at 4°C to separate the serum to be used for biochemical analysis. After blood collection, rats were dissected then the livers were excised and small sections were cut with cold knife. The first liver section was used for the preparation of the liver tissue homogenate to be used for the biochemical tissue analysis. The second liver section was used for the histopathological examination. The third liver section was used for the comet assay.

### Biochemical analysis

#### 1. Determination of liver function enzymes

ALT and AST activities were determined in the sera of rats using Stanbio kit following the method described by **Bergmeyer et al. **[20]. GGT was measured in the sera of rats using Centronic GmbH- Germany Kit following the method described by **Persijn and van der Slik **[21].

#### 2. Determination of hepatic reduced glutathione, lipid peroxidation and antioxidant enzymes

The hepatic GSH content was estimated by the method of **Beutler et al. **[22]. Lipid peroxidation was measured in the liver tissue as MDA level according to the method of **Ruiz-Larrea et al. **[23]. Glutathione reductase (GR) activity was assayed in the liver tissue according to the method of **Erden and Bor **[24]. Glutathione peroxidase (GPx) activity was estimated in the liver tissue by the method of **Paglia and Valentine **[25]. Superoxide dismutase (SOD) activity in the liver tissue was measured by the method of **Nishikimi et al. **[26]. Total proteins were assayed in the liver tissue according to the method of **Bradford **[27].

#### 3. Determination of vascular endothelial growth factor (VEGF)

The measurement of VEGF in the sera of rats using ELISA technique according to the method of **Ferrara **[28] using rat VEGF ELISA kit, ELISA (Dynatech MR 5000, Midland, ON, Canada), Washer (Dynatech MRW, Gaithersburg, MD, USA), Shaker (Varishaker-Incupator, NJ, USA) and Printer (Panasonic Quiet KXP2123, Seri Kembangan, Selangor, Malaysia).

### Comet assay (single cell gel electrophoresis, SCGE)

DNA damage was measured using the comet assay under alkaline conditions and dim indirect light. The SCGE assay was performed essentially as described with some modifications [29]. The liver was excised, washed in saline solution and a small fragment of the liver was transferred to a Petri dish kept on ice. The fragment was washed, minced and suspended into 1 ml cold Hank's balanced salt solution (HBSS) containing 20 mM EDTA and 10% dimethylsulphoxide (DMSO). The fragment was cut into smaller pieces using a disposable microtome razor blade and the solution was aspirated. A fresh mincing solution was added and the liver samples were minced again into finer pieces. The suspension containing isolated cells was transferred to a tube maintained on ice until the preparation of the slides [30]. The quantity of liver cells in the cell suspensions was determined in Giemsa-stained smears. From the liver cell suspension containing approximately 2 × 10^4^-5 × 10^4 ^cells/ml, 5 μl was removed and mixed with 95 μl of 0.5% low melting point agarose; LMPA (in Ca^+2 ^and Mg^+2 ^free PBS) to prepare the final cell-agarose suspension. From the final cell-agarose suspension, 80 μl was spread over the microscope slide (75 × 25 mm glass slides with 19 mm frosted ends, Gibco-BRL), pre-coated with 1% normal melting point agarose; NMPA (Gibco-BRL). The cells were then lysed in freshly prepared buffer containing 2.5 M NaCl, 100 mM EDTA, 10 mM Tris (pH 10.0), 1% Triton X-100 and 10% DMSO for 24 h at 4°C. After lysis, the slides were rinsed three times in deionized water to remove salt and detergent. The slides were placed in a horizontal electrophoresis unit (Cleaver Scientific Ltd, UK) and DNA was allowed to unwind for 20 min in alkaline solution containing 300 mM NaOH and 1 mM EDTA, pH > 13. The DNA was electrophoresed for 20 min at 300 mA and 30 V (0.90 V/cm). The slides were then neutralized with 0.4 m Tris (pH 7.5), fixed for 5 min in absolute alcohol, air-dried and stored at room temperature. Immediately before analysis, the DNA was stained with 50 μl ethidium bromide (20 μg/ml).

### Data scoring and photomicrographs

The fluorescent labeled DNA was visualized (magnification 400x) using an automated fluorescence microscope (Carl Zeiss, Germany) and the images were captured on a computer, equipped with CometScore software (Komet IV). Three parameters were adopted as indicators of DNA damage: tail length (TL in μm),% DNA in comet tail (% DNA in tail) and tail moment (TM in arbitrary units, TM = TL X% DNA in tail).% DNA in tail is the most preferred parameter because it covers a wide range of damage and is linearly related to the break frequency [31].

### Histopathological examination

Tissue specimens from liver were collected from all experimental groups at the end of experiment and fixed in neural buffered formalin 10%, dehydrated in ascending concentration of ethanol, cleared in xylene and embedded in paraffin. Sections 4-5 μm in thickness were prepared and stained with Hematoxylin and Eosin [32].

### Statistical analysis

The data obtained was statistically analyzed using SPSS software package (version 7.5). Hypothesis testing methods included one way analysis of variance (ANOVA) followed by least significant differences (LSD). Values are expressed as mean ± S.D. *P *value ≤ 0.05 was considered significant.

## Results

### Confirmation of the induction of HCC in rats

During the experiment, we dissected four rats from NDEA treated group at the end of 9^th ^week of experiment (i.e the end of 8^th ^week of NDEA administration) and liver was examined histopathologically. The histopathological examination of the liver revealed that HCC was induced.

### Effect of silymarin and EGb on hepatic MDA, GSH and antioxidant enzymes

A significant increase in MDA level was observed in NDEA treated rats compared to control rats (table 1). Prophylactic treatment with either silymarin or EGb for 13 weeks showed a significant protection against NDEA induced lipid peroxidation (table 1). Therapeutic treatment with either silymarin or EGb for 4 weeks offered a significant decrease in MDA level compared to NDEA treated rats (table 1). NDEA administration led to a significant depletion in hepatic GSH content compared with control rats (table 1). Pretreatment and posttreatment with either silymarin or EGb significantly improved hepatic GSH level compared to NDEA group (table 1). The activities of hepatic antioxidant enzymes; GR, GPx and SOD were significantly decreased in NDEA group compared with control animals (table 1). The pretreated and posttreated groups with either silymarin or EGb showed a significant increase in hepatic GR, GPx and SOD activities compared to NDEA intoxicated group (table 1).

**Table 1:** Hepatic MDA, GSH levels, GR, GPx and SOD activities in different experimental groups

*Groups*	*MDA* *(nmol MDA/g tissue)*	*GSH* *(mg/g tissue)*	*GR* (nmol NADPH reacted/min/mg protein)	*GPx* (nmol NADPH reacted/min/mg protein)	*SOD* (nmol NADPH reacted/min/mg protein)
***Control***	11.65 ± 1.10	6.86 ± 1.21	85.94 ± 5.74	102.26 ± 8.43	49.34 ± 3.08
***NDEA***	22.97 ± 2.79 ^a^	0.43 ± 0.10 ^a^	51.94 ± 2.88 ^a^	18.34 ± 3.49 ^a^	5.28 ± 0.82 ^a^
***Pre S.m***	13.89 ± 1.89 ^b^	3.38 ± 0.16 ^b^	72.44 ± 3.91 ^b^	25.26 ± 2.76 ^b^	37.04 ± 4.76 ^b^
***Pre G.b***	14.42 ± 1.56 ^b^	4.54 ± 1.00 ^b^	77.27 ± 13.15 ^b^	36.63 ± 4.10 ^b^	32.28 ± 3.23 ^b^
***Post S.m***	16.74 ± 2.90 ^b,c^	2.36 ± 0.45 ^b,c^	62.51 ± 5.50 ^b,c^	20.00 ± 1.67 ^c^	17.39 ± 2.40 ^b,*c*^
***Post G.b***	17.53 ± 3.32 ^b,d^	3.64 ± 0.57 ^b,d^	61.47 ± 6.42 ^b,d^	29.84 ± 2.92 ^b,d^	20.92 ± 1.77 ^b,d^

Results are given as mean ± S.D. for 6 rats. ***Pre S.m ***= Pretreated silymarin, ***Pre G.b ***= Pretreated EGb, ***Post S.m ***= Posttreated silymarin, ***Post G.b ***= Posttreated EGb. a: significantly different from **control **group at *p *≤ 0.05. b: significantly different from **NDEA **group *at p *≤ 0.05. c: significantly different from ***Pre S.m ***group *at p *≤ 0.05. d: significantly different from ***Pre G.b ***group at *p *≤ 0.05.

### Effect of silymarin and EGb on serum transaminases and gamma glutamyltransferase

The activities of serum ALT, AST and GGT were significantly increased in NDEA treated group compared to control group (table 2). Pretreatment and posttreatment with either silymarin or EGb significantly reduced the elevation in the serum ALT, AST and GGT activities induced by NDEA administration (table 2).

**Table 2:** Effect of silymarin and *Ginkgo biloba *extract on ALT, AST and GGT activities in the sera of rats of different experimental groups

*Groups*	*ALT(U/L)*	*AST(U/L)*	*GGT(U/L)*
***Control***	23.05 ± 1.68	62.47 ± 3.65	31.68 ± 4.38
***NDEA***	75.20 ± 1.52 ^a^	101.22 ± 2.66 ^a^	87.84 ± 10.45 ^a^
***Pre S.m***	41.43 ± 1.13 ^b^	82.27 ± 1.54 ^b^	53.02 ± 4.77 ^b^
***Pre G.b***	36.09 ± 1.24 ^b^	81.62 ± 3.05 ^b^	53.29 ± 7.68 ^b^
***Post S.m***	53.55 ± 2.48 ^b^,^c^	92.23 ± 6.24 ^b^,^c^	68.03 ± 5.36 ^b^,^c^
***Post G.b***	46.97 ± 2.09 ^b^,^d^	95.47 ± 3.71 ^b^,^d^	68.27 ± 8.39 ^b^,^d^

Results are given as mean ± S.D. for 6 rats. ***Pre S.m ***= Pretreated silymarin, ***Pre G.b ***= Pretreated EGb, ***Post S.m ***= Posttreated silymarin, ***Post G.b ***= Posttreated EGb. a: significantly different from **control **group at *p *≤ 0.05. b: significantly different from **NDEA **group at *p *≤ 0.05. c: significantly different from ***Pre S.m ***group at *p *≤ 0.05. d: significantly different from ***Pre G.b ***group at *p *≤ 0.05.

### Effect of silymarin and EGb on serum VEGF

**Figure 2:**
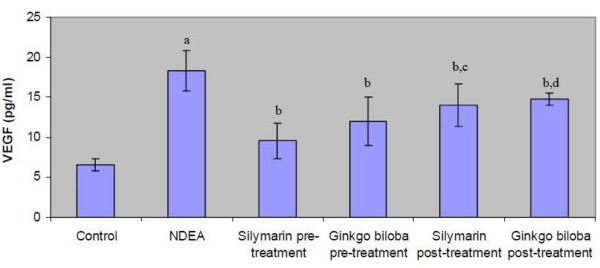
**Serum VEGF level of the rats of different experimental groups**. Results are given as mean ± S.D. for 6 rats. a: significantly different from control group at *p *≤ 0.05. b: significantly different from NDEA group at *p *≤ 0.05. c: significantly different from silymarin pretreated group at *p *≤ 0.05. d: significantly different from *Ginkgo biloba *pretreated group at *p *≤ 0.05.

The serum VEGF level in NDEA group showed the most highly significant elevation compared to control rats (figure 2). All treated groups with either silymarin or EGb showed a significant reduction in serum VEGF level compared to NDEA intoxicated group (figure 2).

### Effect of silymarin and EGb on DNA damage

**Figure 3:**
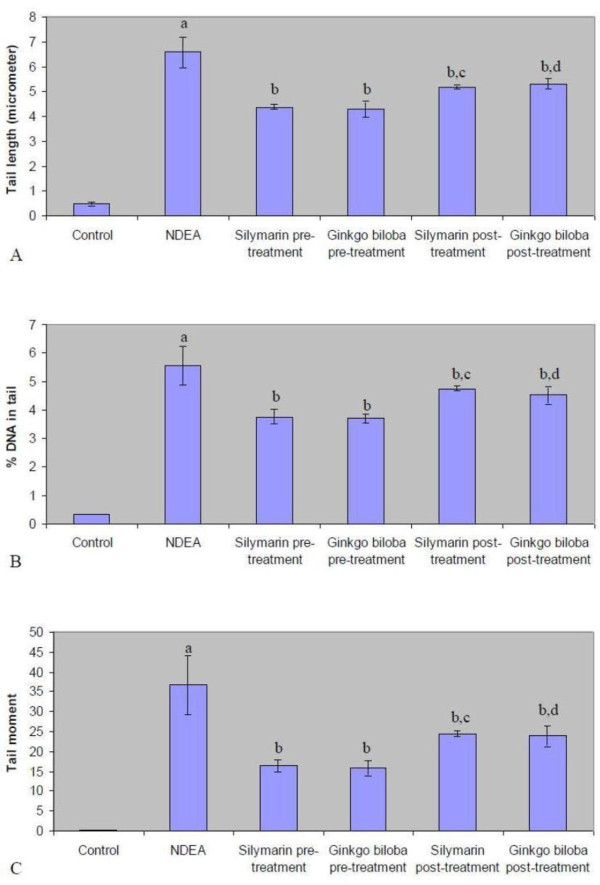
**Comet assay parameters in the hepatic tissue of rats of different experimental groups**. Results are given as mean ± S.D. for 4 rats. a: significantly different from control group at *p *≤ 0.05. b: significantly different from NDEA group at *p *≤ 0.05. c: significantly different from silymarin pretreated group at *p *≤ 0.05. d: significantly different from *Ginkgo biloba *pretreated group at *p *≤ 0.05.

**Figure 4:**
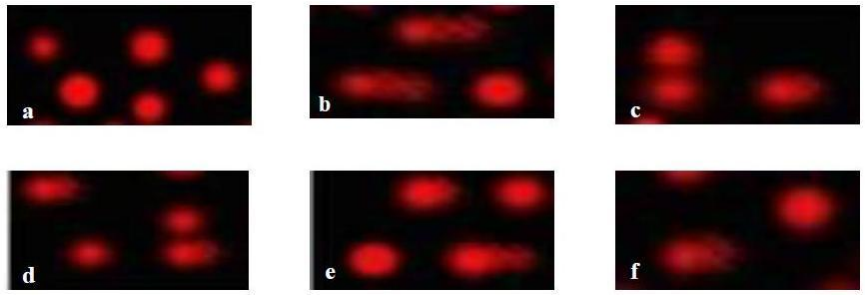
**Photomicrographs of comets in the hepatocytes stained with ethidium bromide in different experimental groups**. Control **(a)**, NDEA **(b)**, Silymarin pretreated **(c)**, EGb pre-treated **(d)**, Silymarin post-treated **(e) **and EGb post-treated **(f) **(400x).

A significant increase in different comet assay parameters (TL,% DNA in tail and TM) has been shown in animals receiving NDEA compared with the negative control animals (figure 3). Prophylactic and therapeutic treatment with either silymarin or EGb significantly reduced NDEA induced DNA damage as indicated by reduction in different comet assay parameters (TL,% DNA in tail and TM) (figure 3). Photomicrographs of comets in the hepatocytes stained with ethidium bromide in different experimental groups are illustrated in figure (4).

### Effect of silymarin and EGb on liver histopathology

**Figure 5:**
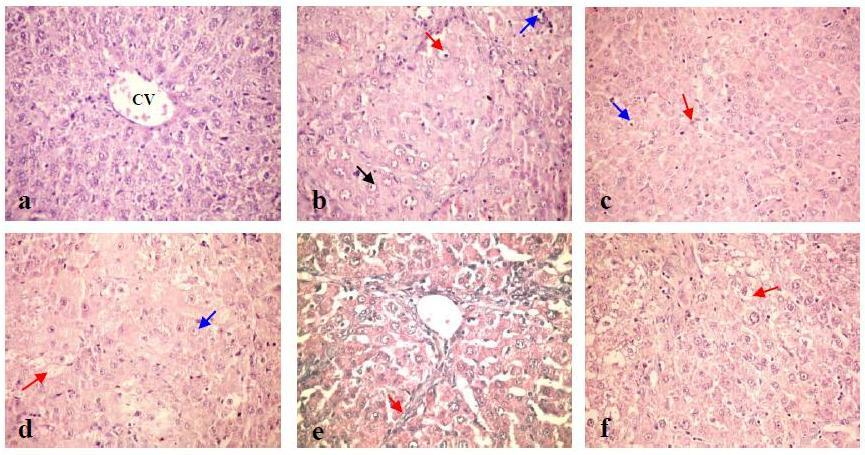
**Histopathological photomicrographs of the liver of rats of different experimental groups**. **(a) **Liver from control group showing normal hepatic architecture with the central vein (CV) lying at the center of the hepatic lobule surrounded by the hepatocytes (H & E stain-X 400). **(b) **Liver from NDEA administrated group showing disorganization of hepatic lobular architecture and obvious cellular damage. The hepatocytes lost their normal shape, lost their arrangement and showed megalocytosis (red arrow), hyperchromatic nuclei (blue arrow) as well as nuclear vesiculation and nuclear prominence (black arrow) (H & E stain-X 400). **(c) **Liver from silymarin pretreated group showing Kupffer cells proliferation (red arrow), minimal nuclear vesiculation and nuclear prominence (blue arrow) (H & E stain-X 400). **(d) **Liver from EGb pretreated group showing hepatocytes with foamy cytoplasm (red arrow), minimal nuclear vesiculation and nuclear prominence (blue arrow) (H & E stain-X 400). **(e) **Liver from silymarin posttreated group showing oval cells proliferation (red arrow) (H & E stain-X 400). **(f) **Liver from EGb posttreated group showing lesser degree of vacuolated hepatocytes (red arrow) (H & E stain-X 400).

Histopathological examination of liver sections from control group revealed normal architecture (figure 5a). While, liver sections of NDEA administered animals revealed well differentiated HCC with apoptosis of hepatocytes, mitotic figures, megalocytosis, foamy cytoplasm and hyperchromatic nuclei (figure 5b). Liver sections of rats pretreated with either silymarin or EGb restored many of normal hepatic architecture with less disarrangement and degeneration of hepatocytes, minimal nuclear vesiculation and nuclear prominence compared with NDEA treated group (figure 5c,d). Meanwhile, liver sections of rats posttreated with either silymarin or EGb revealed slight improvement in the hepatocytes compared with NDEA treated group. Liver sections of rats posttreated with silymarin revealed lesser degree of cytomegalic and vacuolated hepatocytes with minimal nuclear vesiculation and nuclear prominence compared to HCC-induced animals (figure 5e). The proliferation of oval cells had been also observed (figure 5e). Liver sections of rats posttreated with EGb revealed lesser degree of vacuolated hepatocytes and kupffer cells activation compared with NDEA treated group (figure 5f).

## Discussion

N-nitrosodiethylamine, as a well known potent hepatocarcinogenic agent, may be present in tobacco smoke, water, cured and fried meals, cheddar cheese, agricultural chemicals, cosmetics and pharmaceutical products. It is also found in minute concentrations in baby bottle nipples [33]. Metabolism of certain therapeutic drugs is also reported to produce NDEA [34].

Data presented in our investigation indicated that administration of NDEA lead to induction of HCC and augmentation of oxidative stress in livers of NDEA treated rats. NDEA impaired antioxidative defense as indicated by a significant elevation in the level of oxidative stress marker (MDA) and a significant depletion of free radical scavenging antioxidants (GR, GPx, SOD and GSH). The oxidative stress in livers of HCC-induced rats may be attributed to that NDEA is mainly metabolized in the liver by the action of cytochrome p450 enzymes and the reactive metabolites are primarily responsible for its hepatotoxic effects. NDEA is bioactivated to ethyldiazonium ion which alkylates DNA bases to form promutagenic adducts such as O^6^-ethyldeoxyguanosine and O^4 ^and O^6^-ethyldeoxythymidine and these ROS induce oxidative stress and cytotoxicity by damaging biomolecules such as DNA, lipids and proteins [35]. Depletion in GSH level and GSH dependent enzymes, GPx and GR, in NDEA treated rats might be attributed to the reduction in their biosynthesis during hepatocellular damage or their excessive utilization in scavenging the free radicals formed during the metabolism of NDEA. Furthermore, the decreased levels of cellular GSH might have caused a reduction in the activities of GSH dependent enzymes, GPx and GR, as GSH is a vital co-factor for these enzymes [11,36].

Supplementation of the extracts under investigation (silymarin and EGb) to NDEA treated animals effectively modulates the deterioration in the oxidative stress marker, MDA as well as the antioxidant indices, SOD, GR, GPx and GSH implying the beneficial antioxidant abilities of these extracts. Supporting our findings with silymarin, **Ramakrishnan et al. **[37] reported a significant decrease in lipid peroxidation with a significant increase in GSH level as well as an improvement in the activities of the antioxidant enzymes, GR and GPx in the haemolysate and the liver of silymarin treated animals compared to NDEA treated animals. These revealed the anti-lipid peroxidative activity of silymarin and its ability to inhibit free radical generation. The increase in GSH may reduce the DNA-carcinogen interaction by providing a large nucleophilic pool for electrophilic carcinogen (NDEA). GSH neutralizes the electrophilic site by providing SH group and renders the metabolite more water soluble [38]. It has been reported that there is an improvement in the activities of the antioxidant enzymes as NADPH required for the production of GSH is produced by the pentose phosphate pathway [39]. Silymarin has the ability to increase the glucose uptake in the cells which might enhance the levels of glucose uptake by the cells that ultimately serves as fuel for both pentose phosphate pathway and oxidative phosphorylation thereby bringing up the cellular levels of NADPH/NADP^+^. Increasing the levels of NADPH, GR activity also is improved thereby raising the levels of the GSH, the substrate for GPx [40]. Supporting our findings with EGb, **Naik and Panda **[41] reported that EGb significantly increased the activities of free radical scavenging enzymes, SOD, GPx, GR and catalase and the non-enzymatic antioxidant, GSH in CCl_4_-treated rats compared with CCl_4 _treatment alone. *Ginkgo biloba *extract inhibits lipid peroxidation by scavenging free radicals and ROS maintaining the integrity and permeability of cell membranes thereby protecting cells and tissues against oxidative stress induced by the free radicals [42]. These beneficial effects of EGb could be explained not only by its antioxidant properties, but also by its ability to inhibit the main cytochrome P450 isoenzyme 2E1 (CYP2E1) that metabolizes NDEA and CCl_4 _inducing ROS generation and lipid peroxidation [35,43].

Researchers recognize that tumor growth is angiogenesis-dependent and every increment of tumor growth requires an increment of vascular growth. Tumors lacking angiogenesis remain dormant indefinitely and rapid logarithmic growth follows the acquisition of a blood supply. Therefore, many researchers show great interests in identifying and modulating antiangiogenic pathways and developing antiangiogenic drugs for therapeutic purposes [14,44]. VEGF is one of the most important angiogenic cytokines. The overexpression of VEGF has been shown to enhance tumor growth and its expression correlates with poor prognosis in several types of tumors including HCC which is a typical hypervascular tumor [45]. Data in the present study showed that the serum VEGF level in NDEA treated rats was significantly elevated compared to the normal healthy rats. **Liu et al. **[46] reported that the expression of VEGF in NDEA treated group was remarkably elevated and no positive expression of VEGF was found in the negative control group. These results might be attributed to the high angiogenic activity in NDEA-induced hepatocarcinoma rats, in addition to the increase of nitric oxide activity in NDEA treated animals compared to the negative control animals, thus enhancing the angiogenesis by stimulating the synthesis of VEGF [47]. **Turlin et al. **[48] also reported an overexpression of VEGF in hepatic foci and tumors in NDEA treated animals, this high expression was associated with breakdown of the vascular wall by tumor cells. Since VEGF can act as a permeability factor, this suggests a relationship between VEGF expression and invasion of the vascular wall. Indeed, VEGF may induce a breakdown of vascular walls by inducing protease synthesis which may result in damaging vascular structures [49].

The current investigation revealed a significant decrease in serum VEGF level in silymarin supplemented rats compared to NDEA treated animals. Our results are in accordance with **Jiang et al. **[50] who reported that silymarin has antiangiogenic activity that may contribute critically to its cancer chemopreventive potential. The antiangiogenic effect of silymarin might be attributed to its rapid inhibitory action on the secretion of the primary angiogenic cytokine VEGF by the cancer epithelial cells. Also, silymarin inhibited endothelial matrix metalloproteinase-2 (MMP-2) secretion and expression, thus silymarin may provide an inhibitory mechanism on angiogenesis independent of its effect on VEGF [51]. Additionally, silybin (the main active constituent of silymarin) decreased the expression of hypoxia inducible factor-1α (HIF-1α) and inducible nitric oxide synthase (iNOS) that induce angiogenesis [52]. The current investigation also revealed a significant decrease in serum VEGF level in EGb supplemented rats compared to NDEA treated animals. Recent study demonstrated the requirement for the extracellular signal-regulated kinase (ERK) in growth factor induced angiogenesis. It has been shown that EGb reduces ERK phosphorylation induced by the growth factors VEGF and fibroblast growth factor (FGF) in an endothelial cell model leading to the suggestion that EGb inhibits angiogenesis via ERK inhibition as EGb activates protein tyrosine phosphatases [53]. It has been also shown that EGb inhibits the ''respiratory (oxidative) burst'' that results from the activation of human neutrophils, an action that could be associated with antiangiogenic and antitumorigenic activities because activated human neutrophils have been shown to be directly angiogenic via their release of VEGF and hepatocyte growth factor/scatter factor, two cytokines that have potent proangiogenic properties [54,55].

The comet assay (SCGE) is a rapid, sensitive and versatile method for the quantification of DNA damage in the individual cells both *in vitro *and *in vivo *[56]. In the comet assay, cells with damaged DNA displayed increased migration of DNA fragments (comet tail) from the nucleoid (comet head) which may also be a feature of DNA fragmentation associated with the necrotic/apoptotic death process [57]. The current investigation demonstrated a highly significant increase in the comet assay parameters (TL,% DNA in tail and TM) in NDEA treated rats compared to the healthy control rats. This is in consistent with the previous results of **Horst et al. **[58] who reported NDEA-induced DNA damage indicated by the markedly increased length of the comets (head + tail) in the liver cells of rats administrated NDEA compared to the negative control rats. The increment of such parameters may be due to that the enzymes of CYP2E1 subfamily play a role in the biotransformation of a range of compounds, including NDEA, producing the promutagenic DNA lesions which play an important role in DNA damage and induction of hepatocarcinogenesis [35].

Silymarin supplementation in our study was effective in suppressing DNA damage induced by NDEA showing a significant decrease in the comet assay parameters (TL,% DNA in tail and TM) compared to NDEA treated group. These results were consistent with **Saravanan and Pugalendi **[59] who reported that the coadministration of silymarin with alcohol significantly decreased the DNA damage when compared with alcohol treated rats. This protective effect of silymarin can be explained by its ability to scavenge the free radicals before they cause damage to nuclear DNA [60]. *Ginkgo biloba *extract supplementation in our study significantly diminished DNA damage caused by NDEA as indicated by a significant decrease in the comet assay parameters (TL,% DNA in tail and TM) compared to NDEA treated group. This was attributed to that EGb is cytoprotective; it is able to permeate cell membranes and exert its protective action on the nuclear DNA and also cytoplasmic components [61]. **Keles et al. **[62] reported that pretreatment with EGb clearly diminished 8-hydroxydeoxyguanosine (8-OHdG) formation in DNA in the hepatic tissue of rats that had undergone liver ischaemia-reperfusion (IR). This protective effect of EGb has been attributed to its high free radical scavenging ability. **Min and Ebeler **[63] suggested the potential of quercetin (the major component of flavonoid glycosides of EGb) to protect against cancer by inhibiting oxidative DNA damage as well as by enhancing DNA repair after oxidant challenge in Caco-2 cells (colon cells).

Serum AST and ALT are the most sensitive markers employed in the diagnosis of hepatic damage because they are cytoplasmic in location and hence released into the circulation after cellular damage. Analysis of these marker enzymes reflects mechanisms of cellular damage, subsequent release of proteins, their extracellular turnover and mechanisms of neoplastic processes [64]. GGT is an enzyme embedded in the hepatocyte plasma membrane mainly in the canalicular domain and its liberation into serum indicates damage to the cell, thus injury to the liver [65]. The present study demonstrated that activities of ALT, AST and GGT in the sera of NDEA treated rats were markedly elevated compared to the normal healthy control rats. This increment is due to the production of free radicals during NDEA metabolism, thus damaging the hepatocellular membrane. As a result, these cytoplasmic enzymes are released into the systemic circulation.

As seen in the present study, treatment with silymarin significantly reduced serum ALT, AST and GGT activities compared to NDEA treated animals. This might be attributed to the ability of silymarin to scavenge the free radicals, thus preventing the hepatocellular damage caused by NDEA, thereby suppressing the leakage of enzymes through plasma membranes [11]. Supplementation of EGb to HHC-induced animals effectively lowers the high serum activities of AST, ALT and GGT produced by NDEA. This hepatoprotective effect of EGb could be due to a modifying influence on the biotransformation/detoxification of NDEA, thus reducing its liver toxicity [66].

The biochemical findings in our study were supported by the histopathological examination of the liver tissue of experimental animals. The histopathological observation of the livers of NDEA treated rats revealed well differentiated HCC with disorganization of hepatic lobular architecture and obvious cellular damage. The hepatocytes exhibited megalocytosis, foamy cytoplasm and hyperchromatic nuclei. This is in agreement with the results of **Gupta et al. **[67] who reported that the histological examination of the liver tissue of rats treated with NDEA revealed vacuolization, loss of normal hepatocellular architecture and the presence of pycnotic nuclei. This may be attributed to that NDEA is primarily metabolized in the liver and reactive metabolites generated thereby are known to damage hepatocytes.

Histopathology of the liver tissue of rats pretreated with silymarin revealed an improvement in the hepatocytes exhibiting less disarrangement and degeneration of hepatocytes compared to NDEA treated rats. Liver sections of rats posttreated with silymarin revealed a slight improvement in the hepatocytes exhibiting lesser degree of cytomegalic, vacuolated hepatocytes with minimal nuclear vesiculation and prominence compared with NDEA treated group. This improvement may be attributed to that silymarin can efficiently reduce intracellular ROS levels of hepatocytes, thus preventing oxidative stress-induced cellular damage. Furthermore, hepatic cell proliferation was found to be stimulated after silymarin treatment suggesting that enhanced liver regeneration may help replace the damaged liver cells [12]. Liver sections of rats pretreated with EGb revealed an improvement in the hepatocytes exhibiting less disarrangement and degeneration of hepatocytes compared with NDEA treated group. The liver sections of rats posttreated with EGb revealed a slight improvement in the hepatocytes exhibiting lesser degree of vacuolated hepatocytes and kupffer cells activation compared with NDEA treated group. These beneficial effects of EGb can be partially explained by its antioxidant properties and by inhibition of CYP2E1 that metabolizes NDEA in the liver [35].

Our study revealed that pretreatment with silymarin and *Ginkgo biloba *extract presented more protection of liver against NDEA induced damage than posttreatment with these extracts. Therefore, the chemopreventive activities of silymarin and *Ginkgo biloba *extract during initiation stage are more effective than post-initiation stage of rat hepatocarcinogenesis induced by NDEA. In conclusion, this study demonstrated that silymarin and *Ginkgo biloba *extract have been found to possess a beneficial protective effect against NDEA-induced hepatocarcinogenesis through their antioxidant, antigenotoxic and antiangiogenic activities. From this, we can hypothesize that these extracts are strong candidates as chemopreventive agents for liver cancer.

## Competing interests

The authors declare that they have no competing interests.

## Authors' contributions

HOE conceived of the study, developed the study protocol, supervised the experimental work and corrected the manuscript. NSM participated in the protocol writing, supervised samples' analysis and helped to draft the manuscript. MSS participated in the protocol writing, designed the experiments, supervised samples' analysis and helped to draft the manuscript. KAA was responsible for the histopathological part of the study. MMA carried out all the experimental work, performed the statistical analysis and contributed to the writing of the manuscript. All authors read and approved the final manuscript.
